# Editor's Note

**Published:** 1988-02

**Authors:** 


**Editor's note**

In reply to the comments offered in the last paragraph by the authors of the letter, one of whom is his predecessor, the editor acknowledges his lapse from the highest editorial standards in not obtaining a bibliography. Regarding the suggestion that he should have sought advice from 'expert referees' he would like to say that the paper had already been delivered to a meeting of the society which was well attended; his record of the subsequent discussion does not contain any note of authoritative criticism of the views now at issue. The editor asked Dr Seale after the meeting if he would provide a script for publication, he replied by giving him a copy of his memorandum submitted to the Select Committee on Health and Social Services of the House of Commons, on which his talk was based. Your editor abbreviated it and Dr Seale approved this version. Dr Seale is a senior consultant venereologist, formerly at St Thomas hospital and has made a deep study of the problems of AIDS, he is an expert in his own right, his writings on AIDS have been widely published in many journals including The Journal of the Royal Society of Medicine, Nature, The Veterinary Record etc. Having been presented to the House of Commons his paper was already in the public domain, and it is of interest to learn of evidence presented to our legislators. Dr Seale is perhaps a Jeremiah, he is deeply troubled that the gravity of the situation and the need for effective action is not being faced. If his views are controversial, publication of them allows those who disagree to express their contrary opinions which the editor is also glad to publish and for which he thanks them. He hopes that Dr Seale will respond.

